# Time-Varying Insomnia Symptoms and Incidence of Cognitive Impairment and Dementia among Older US Adults

**DOI:** 10.3390/ijerph18010351

**Published:** 2021-01-05

**Authors:** Nicholas V. Resciniti, Valerie Yelverton, Bezawit E. Kase, Jiajia Zhang, Matthew C. Lohman

**Affiliations:** 1 Department of Epidemiology and Biostatistics, Arnold School of Public Health, University of South Carolina, Columbia, SC 29201, USA; bkase@email.sc.edu (B.E.K.); JZHANG@mailbox.sc.edu (J.Z.); LOHMANM@mailbox.sc.edu (M.C.L.); 2 Department of Health Services Policy & Management, Arnold School of Public Health, University of South Carolina, Columbia, SC 292901, USA; VYELVERT@mailbox.sc.edu

**Keywords:** dementia, cognitive impairment, time-varying, insomnia, older adults, sleep

## Abstract

There is conflicting evidence regarding the association between insomnia and the onset of mild cognitive impairment (MCI) or dementia. This study aimed to evaluate if time-varying insomnia is associated with the development of MCI and dementia. Data from the Health and Retirement Study (*n* = 13,833) from 2002 to 2014 were used (59.4% female). The Brief Insomnia Questionnaire was used to identify insomnia symptoms which were compiled in an insomnia severity index, ranging from 0 to 4. In analysis, participants’ symptoms could vary from wave-to-wave. Dementia was defined using results from the Health and Retirement Study (HRS) global cognitive assessment tool. Respondents were classified as either having dementia, MCI, or being cognitively healthy. Cox proportional hazards models with time-dependent exposure using the counting process (start-stop time) were used for analysis. For each one-unit increase in the insomnia symptom index, there was a 5-percent greater hazard of MCI (HR = 1.05; 95% CI: 1.04–1.06) and dementia (HR = 1.05; 95% CI: 1.03–1.05), after fully adjusting. Using a nationally representative sample of adults age 51 and older, this study found that time-varying insomnia symptoms are associated with risk of MCI and dementia. This highlights the importance of identifying sleep disturbances and their change over time as potentially important risk factors for MCI and dementia.

## 1. Introduction

Dementia is a progressive neurodegenerative disorder affecting individuals’ brain function, memory, and activities of daily living [1]. The prevalence of dementia is expected to triple in the next 40 years, leading to an estimated 14 million people with dementia by 2060 [2]. The Alzheimer’s Association estimates the total direct medical expenditures related to Alzheimer’s disease-related dementias (ADRD) will increase from $236 billion in 2016 to over $1 trillion in 2050 [3].

Mild cognitive impairment (MCI) is defined as self- or informant-reported functional impairment or impaired test performance on neuropsychological measures that does not reach the severity of dementia [4]. MCI can be a precursor of dementia [5,6], with over 30 percent of individuals diagnosed with MCI progressing to dementia within five years [1]. MCI is estimated to affect 15–20 percent of older adults in the US [1,7,8,9]. While there are several subtypes of MCI with diverse etiology, there is still much debate regarding the pathogenesis of MCI and dementia [5]. Previous research has identified several modifiable and immutable factors contributing to an individual’s risk for dementia and MCI including socio-demographics, comorbidities, family history, and lifestyle factors [1,5,8,10,11,12].

Growing evidence has linked sleep disturbances and insomnia to greater risk of MCI and dementia [13,14,15,16,17,18,19,20,21,22,23,24]. In particular, the association between insomnia, which includes difficulty falling asleep, awakening during the night, early-awakening without being able to fall asleep again, and non-restorative sleep [25], and cognitive decline has received significant research attention. Results from several studies indicate that individuals with insomnia are more likely to have clinically significant alterations in attention and episodic memory and more likely to develop dementia [26,27,28]. However, very little is known regarding the frequency or level of insomnia that would result in MCI and dementia. Published studies examining the associations between insomnia and MCI or dementia have predominantly assumed insomnia symptoms to be stagnant over time or have not considered the severity or type of insomnia symptoms [14,17,29,30,31,32]. These assumptions may be especially tenuous among older adult populations, as previous research shows that insomnia symptoms vary significantly over time and frequently increase as individuals get older [28]. Further, research suggests that measuring insomnia at only one timepoint may lead to biased estimates of the associations with MCI and dementia, which may have important implications for public health and clinical approaches to addressing cognitive health [31]. Thus, to understand the role of insomnia in the development of MCI and dementia, it is necessary to distinguish chronic insomnia disorder from presence of insomnia symptoms at a single point in time.

Given the limitations of previous analyses, this study examined the dynamic effect of changes in insomnia on cognitive decline and dementia over time. Our primary aim was to estimate the association between time-varying insomnia and MCI or dementia using a nationally representative cohort of US adults age 51 and older from 2002 to 2014. We hypothesized that time-varying insomnia symptoms would be associated with greater risk of incident MCI and dementia.

## 2. Materials and Methods

### 2.1. Database

This study used data from the Health and Retirement Study (HRS), a biennial, population-based survey of older adults in the United States conducted by the University of Michigan and sponsored by the National Institute on Aging (NIA U01AG009740). HRS sample collection is based on a multi-stage probability design meant to produce a nationally representative sample of adults over the age of 50 [33]. Thirty-five sample weights are provided to account for the complex sampling design and to make inferences regarding the general US population. Respondents are re-interviewed every two years and asked questions related to health status, healthcare utilization, cognition, and demographics [33]. The present study used data from seven HRS waves (2002 to 2014). Of individuals who completed the HRS core survey during the study period, 18,166 adults age 51 and older provided data on their cognitive status and insomnia symptoms at baseline. We excluded respondents who were found to have MCI (*n* = 2996) or dementia (*n* = 1337) at baseline leading to the final analytic sample of 13,833 participants who were cognitively healthy at baseline.

### 2.2. Outcome Variables

MCI and dementia were assessed at each wave of HRS using a global test of several cognitive domains, such as working memory, word recall, language, and orientation; the internal consistency reliability coefficient was about 0.65 [34]. Scores were summed to a composite score that ranged from 0 to 35 [34]. To determine MCI and dementia, we used a previously validated classification approach developed using HRS data [35]. The orientation and language domains were first excluded from the full HRS cognitive score, yielding a composite score ranging from 0 to 27 [35]. Respondents who scored 0 to 6 were classified as having dementia, those who scored from 7 to 11 were classified as being cognitively impaired with no dementia (MCI), and respondents scoring from 12 to 27 were considered not cognitively impaired.

### 2.3. Exposure Variable

Insomnia symptoms were assessed using the Adapted Brief Insomnia Questionnaire (BIQ) [36]. Trouble falling asleep, waking up during the night, waking up too early and not being able to fall asleep again most of the time and feeling really rested when you wake up in the morning rarely or never were considered to be insomnia symptoms. Insomnia was assessed as a severity score from 0 (no symptoms) to 4 (the most symptoms) [36,37].

### 2.4. Covariate Variables

Demographic characteristics considered for model adjustment were baseline (wave 2002) age, gender, race, and education. A baseline chronic disease index was calculated by summing indicators of high blood pressure, diabetes, cancer, lung disease, heart disease, stroke, psychiatric problems, and arthritis. Additional baseline health information used in adjustment were body mass index (BMI), smoking status, and number of alcoholic drinks consumed when drinking. Model covariates were selected based on literature review and an empirical confounder assessment. If adjustment for a potential confounder produced at least a 10% change in estimate compared to the crude association, it was kept in the final model.

### 2.5. Data Analysis

Descriptive statistics of the baseline sample were calculated with percent and means for categorical and continuous variables, respectively. Frequencies of self-reported insomnia symptoms and the change from wave-to-wave were calculated and depicted visually using a Sankey diagram. Adjusted and unadjusted Cox proportional hazards models with time-dependent exposure were used to estimate the associations between insomnia and subsequent incidence of MCI or dementia. Specifically, the counting process model, an extension of the Cox model, was applied to account for the longitudinal information on insomnia, where the follow-up period was divided into intervals (start and stop time) based on each wave [38]. Lastly, each model was stratified by baseline age categories (51–64 and 65 and older; effect modification results were not presented since effect modification by age was not significant) to understand if there was effect modification at baseline by age. HRS analytic weights and stratification variables were applied to all hazard models to account for the complex sample design. All analyses were completed with SAS (Statistical Analysis System) version 9.4 (SAS institute, Cary, NC, USA).

## 3. Results

### 3.1. Study Sample Characteristics

**Figure 1:**
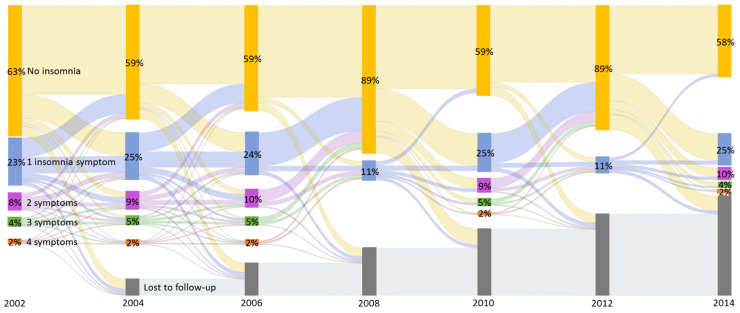
Fluctuation of self-reported insomnia symptoms over time (2002–2014). Different colors indicate the classification of information (yellow: no or 0 self-reported insomnia symptoms; blue = 1 self-reported insomnia symptom; purple = 2 self-reported insomnia symptoms; green = 3 self-reported insomnia symptoms; orange = 4 self-reported insomnia symptoms). Classification of insomnia symptoms is limited to 0 or 1 insomnia symptom(s) in 2008 and 2012. Changes of self-reported insomnia symptoms are represented by curved lines. Data source: Health and Retirement Study (2002–2014).

The analytic sample included 13,833 participants without MCI or dementia at baseline. The mean age of participants was 66.41 years (SD = 9.52 years), and 59% were females. A majority of participants were white (86%) and 17% had less than high school education. The baseline mean insomnia severity score was 0.58 with a SD of 0.92 (see Table 1). The Sankey diagram (Figure 1) shows that the percentages of each insomnia symptom category at each wave were consistent in the period 2002–2006, 2010, and 2014; however, there was substantial fluctuation of reported insomnia symptoms by individuals from wave-to-wave. The fluctuation of symptoms suggests that self-reported insomnia symptoms are time-varying.

### 3.2. Cox Proportional Hazards Model with Time-Varying Insomnia

Table 2 shows the unadjusted and adjusted hazard ratios between time-varying insomnia on MCI and dementia. Time-varying insomnia was significantly associated with both MCI and dementia in both adjusted and unadjusted models. Each one-unit increase in the insomnia symptom index was associated with a 5 percent greater hazard of MCI (HR = 1.05; 95% CI: 1.04–1.06), after controlling for baseline age, race, gender, education, BMI, smoking status, drinking, and the chronic disease index. Each one-unit increase in the insomnia symptom index was associated with a 5 percent greater hazard of dementia (HR = 1.05; 95% CI: 1.03–1.05), after controlling for baseline race, gender, education, BMI, smoking status, drinking, and the chronic disease index. Further stratified analyses according to baseline age were performed but did not find significant differences in the associations between insomnia and MCI or dementia by age (results not presented).

## 4. Discussion

Using a nationally representative sample of adults age 51 and older, this study found that time-varying insomnia severity was associated with a greater likelihood of developing MCI and dementia over time. In support of our hypothesis, over 12 years of follow-up, results suggested that for each additional insomnia symptom, there was a 5 percent greater hazard of incident MCI (HR = 1.05; 95% CI: 1.04–1.06) and dementia (HR = 1.05; 95% CI: 1.03–1.06), after adjusting for baseline gender, race, education, BMI, smoking status, drinking status, and chronic disease index.

Previous research regarding the associations between insomnia and MCI/dementia are mixed and sometimes contradictory. For instance, Hung et al. (2018) found that insomnia was associated with a 2.14 times greater dementia risk [28]. Conversely, Lysen et al. (2018) found that sleep quality was not associated with incident dementia (HR: 0.91; 95% CI: 0.82–1.02) [39]; however, these studies used different sleep measures and different study designs and did not account for time-varying insomnia symptoms, which may explain inconsistent results. Our findings are consistent with a positive association between insomnia and greater risk of incident cognitive decline and dementia. The degree of risk is similar to that found in a meta-analysis by Xu et al. (2020) [23].

Differences between our study and previously conducted studies with different or null findings could be related to several methodologic and sample differences. Our study used a longitudinal study design with time-varying insomnia, while other studies have used cross-sectional [23] or case-control [28] study design or assessed insomnia only at baseline [39]. Additionally, we considered insomnia symptoms on a continuous scale while other studies used clinical diagnosis [28] or categorical insomnia definitions [23]. Consistent with previous research, our results demonstrate that insomnia symptoms vary significantly over time [40,41]. The use of time-invariant insomnia symptoms in analysis may thus fail to distinguish the effects of chronic insomnia from more transient insomnia episodes, for instance due to illness or use of medications. Previous research shows that a significant proportion of people with transient insomnia might not develop chronic insomnia and the consequences of transient insomnia may be subtle [42]. In contrast, chronic insomnia has been associated with a wide range of deleterious health consequences including an increased risk of hypertension, diabetes, obesity, depression, heart attack, and stroke, [28,43], which are also related to risk of cognitive decline and dementia. Information about the duration and changing severity of insomnia may thus provide more information about the cumulative effects of insomnia, both directly and indirectly, on cognition.

Several potential mechanisms have been proposed to explain how insomnia may increase risk of MCI and dementia. First, poor sleep—as defined by insomnia, sleep duration, sleep quality, intake of hypnotics, and sleep-disorder related to breathing—has been shown to be associated with neuronal degradation, structural changes of the brain and lower brain volumes [44,45]. A recent review by Joo (2015) found that insomnia may be associated with abnormal brain structures in the frontal cortex, hippocampus, and anterior cingulate cortex, areas important for memory, executive functions and other cognitive domains. These changes were due to a decrease in gray matter volumes in key regions associated with cognitive function [44]. More specifically, the researchers found evidence of greater atrophy among those with insomnia compared to controls in the CA2-4-DG region of the hippocampus, which was associated with impairment in verbal information processing, verbal memory, verbal processing, and visual memory [45]. Additional studies have found that insomnia symptoms in aging populations are independently associated with neuronal loss in the hippocampus [46]. During healthy sleep, the human brain clears amyloid-beta (Aβ) peptide accumulated during wake phases via the glymphatic pathway [47,48]. Sleep disturbances may thus interrupt or hamper this process resulting in an accumulation of Aβ in the brain, a hallmark of AD pathogenesis [49]. Alternatively, some research suggests that there may be a bidirectional association between sleep disturbances, MCI and dementia [49,50,51]. This bidirectional association may suggest that preclinical MCI or dementia may be influencing the development of insomnia followed by the development of detectable cognitive problems. Further, several modifiable and immutable factors, such as socio-demographics, comorbidities, family history, and lifestyle factors, are associated with insomnia, and over time, can lead to cognitive problems, including dementia and MCI [1,5,8,10,11,12].

The current study has many strengths, including a large sample size, 12-year follow-up, and use of a representative sample of adults age 51 and over in the United States; however, there are some limitations to note. First, insomnia symptoms were self-reported from the BIQ questionnaire and were compiled into an unweighted linear symptom index assuming equal weighting of all symptoms. Additionally, we were unable to confirm a clinical diagnosis of insomnia. Second, both outcome variables relied on cognitive and neuropsychological tests. Using imaging methods and/or clinical diagnosis of MCI and dementia could have led to different estimates of the association with insomnia. Last, this study did not differentiate between types of dementias which and cannot be interpreted in the light of specific forms of dementia, just an overall assessment. Future research should focus on understanding how time-varying insomnia is differentially associated with MCI and dementia compared to baseline or time-invariant insomnia. Additionally, it should be understood whether time-variant insomnia is associated with structural brain changes similar to those found in previous research.

## 5. Conclusions

In conclusion, this study showed that participants with more insomnia symptoms had an increased risk of MCI and dementia. The current results, in conjunction with other studies, highlight the importance of identifying sleep disturbances as a potentially important risk factor for the development of MCI and dementia. It is especially important for primary care providers to assess their patients’ risk of sleep problems, as they may be able to intervene to prevent or delay the onset of MCI or dementia through lifestyle changes (e.g., more exercise or less coffee and alcohol) and behavioral therapy. Future studies should identify the association between fluctuating insomnia symptoms and specific forms of dementia.

## Figures and Tables

**Table 1:** Baseline sample characteristics.

Baseline Characteristics	Total Sample *N* = 13,833
Baseline insomnia (SD)	0.58 (0.92)
Age mean (SD)	66.41 (9.52)
Gender (% Female)	59.37%
Race (% White)	86.29%
Education (% Less than high school)	17.25%
BMI mean (SD)	27.38 (5.30)
Drinks per day (SD)	0.63 (0.01)
Smoking status (%Smoker)	14.13
Chronic disease Index (% No Chronic illness)	19.45

Note: Chronic disease index includes high blood pressure, diabetes, cancer, lung disease, heart disease, stroke, psychiatric problems, and arthritis.

**Table 2:** Unadjusted and adjusted hazard ratios between time-varying insomnia, mild cognitive impairment, and dementia.

		Mild Cognitive Impairment		Dementia	
		Unadjusted	Adjusted *	Unadjusted	Adjusted *
Insomnia		1.10 ^ (1.09–1.11)	1.05 ^ (1.04–1.06)	1.09 ^ (1.07–1.10)	1.05 ^ (1.03–1.06)
Age (Years)			1.06 ^ (1.06–1.06)		1.11 ^ (1.11–1.11)
Education					
	Less than High School		3.60 ^ (3.59–3.60)		2.67 ^ (2.66–2.68)
	GED		2.42 ^ (2.42–2.43)		1.84 ^ (1.83–1.84)
	High School Graduate		2.03 ^ (2.02–2.03)		1.48 ^ (1.47–1.48)
	Some College		1.53 ^ (1.53–1.53)		1.19 ^ (1.19–1.20)
	College and Above		Ref		Ref
Race	White		Ref		Ref
	Black		1.95 ^ (1.95–1.95)		2.09 ^ (2.08–2.10)
	Other		1.62 ^ (1.61–1.62)		1.61 ^ (1.60–1.62)
Gender	Male		Ref		Ref
	Female		0.81 ^ (0.81–0.81)		0.98 ^ (0.98–0.98)
Smoking	No		Ref		Ref
	Yes		1.25 ^ (1.24–1.25)		1.36 ^ (1.36–1.37)
BMI			1.00 (0.99–1.00)		0.98 ^ (0.98–0.98)
Number of Chronic Diseases			1.08 ^ (1.08–1.08)		1.59 ^ (1.59–1.59)
Number of Drinks			0.96 ^ (0.96–0.96)		0.91 ^ (0.91–0.92)

Note: * adjusted for baseline age, gender, race, education, body mass index, drinking, smoking status, and chronic disease index; ^ *p*-value < 0.05.

## Data Availability

Publicly available datasets were analyzed in this study. This data can be found here: https://hrs.isr.umich.edu/data-products.
